# The Postpartum Depressive State in Relation to Perceived Rearing: A Prospective Cohort Study

**DOI:** 10.1371/journal.pone.0050220

**Published:** 2012-11-21

**Authors:** Norika Hayakawa, Takayoshi Koide, Takashi Okada, Satomi Murase, Branko Aleksic, Kaori Furumura, Tomoko Shiino, Yukako Nakamura, Ai Tamaji, Naoko Ishikawa, Harue Ohoka, Hinako Usui, Naomi Banno, Tokiko Morita, Setsuko Goto, Atsuko Kanai, Tomoko Masuda, Norio Ozaki

**Affiliations:** 1 Department of Psychiatry, Nagoya University Graduate School of Medicine, Nagoya, Japan; 2 Faculty of Policy Studies, Nanzan University, Seto, Japan; 3 Liaison Medical Marunouchi, Nagoya, Japan; 4 Sugiyama Jogakuen University, Nagoya, Japan; 5 Graduate School of Education and Human Development, Nagoya University, Nagoya, Japan; 6 Graduate School of Law, Nagoya University, Nagoya, Japan; University of South Florida, United States of America

## Abstract

**Background:**

The relationship between perceived rearing and the postpartum depressive state remains unclear. We aimed to examine whether perceived rearing is a risk factor for postpartum depression as measured by the Edinburgh Postnatal Depression Scale (EPDS), and whether the score of perceived rearing is affected by depressive mood (the state dependency of perceived rearing).

**Methods:**

Pregnant women (n = 448, mean age 31.8±4.2 years) completed the EPDS as a measure of depressive state in early pregnancy (T1), late pregnancy (around 36 weeks), and at 1 month postpartum (T2), and the Parental Bonding Instrument (PBI) at T1 as a measure of perceived rearing. Changes in the EPDS and the PBI scores from T1 to T2 were compared between the non depressive (ND) group and the postpartum depressive (PD) group.

**Results:**

There were no significant differences in any PBI category for perceived rearing between the ND and PD groups at T1. EPDS scores did not change significantly from T1 to T2 in the ND group but increased significantly in the PD group. The PBI maternal care score increased significantly in the ND group (p<0.01), while decreasing in the PD group (p<0.05). Additionally, in both the ND and PD groups, significant negative correlation was observed regarding change in the EPDS and PBI maternal care scores from T1 to T2 (r = −0.28, p = 0.013).

**Conclusions:**

The present study suggests that perceived rearing is not a strong risk factor for postpartum depression as measured by the EPDS. Furthermore, the results indicated the state dependency of the PBI maternal care score.

## Introduction

Postpartum depression, which is observed in about 13% of postpartum women, is defined as a depressive disorder occurring within four weeks after delivery [1]. Postpartum depression not only leads to substantial impairment of the patient’s daily life, but also has huge impact on the patient’s family members including promotion of depressive tendencies in the husband, abuse or neglect of the child [2], and delayed cognitive development or increase in psychopathological problems in the child [3], [4], [5]. Consequently, identification of the risk factors in postpartum depression is important, so that appropriate preventive and early intervention measures may be taken for pregnant and postpartum women.

There have been recent studies attempting to establish the relationship between postpartum depression and perceived rearing (own memories of being raised) in women. For example, Boyce *et al.* [6] have reported that the onset of postpartum depression is associated with parental rearing styles manifested in the form of low care or high overprotection, as well as high interpersonal sensitivity regarding the relationship with the husband. Similar findings by Matthey *et al.* [7] revealed that the onset of postpartum depression 4 months after delivery in patients showing no signs of depression during pregnancy depended on the relationship with their partners, the high interpersonal sensitivity, and quality of early parent-child relationships characterized by low care and high overprotection. However, in their study, depression at 12 months after delivery was not linked to early relationships with parents, but only to relationships with the partner. On the other hand, McMahon *et al.* [8] have reported low maternal care as a cause of persistent depressive symptoms even at 12 months after delivery.

Historically, Gotlib *et al.* [9], examining the influence of depressed mood in postpartum depression on perceived rearing using the Parental Bonding Instrument (PBI), concluded that perceived rearing, evaluated retrospectively, was independent of the state of depression at the time of evaluation. It was also reported that scale scores regarding perceived rearing had long-term stability and that scores were not skewed by mood states at the time of evaluation [10], [11], [12], [13]. On the contrary, a more recent report [14] suggests that depressed mood might influence the evaluation of perceived rearing, indicating the relationship between depression and perceived rearing is as yet unclear.

A prospective cohort study was performed in women during pregnancy and the postpartum to examine the prevalence and course of depressive mood occurring during these periods, and to identify the risk factors involved in the development of depression as measured by the Edinburgh Postnatal Depression Scale (EPDS) [15]. The results indicated that the clinical course of postpartum depressive state could be classified into four patterns based on scores of the Japanese version of the EPDS in early pregnancy, late pregnancy, and at 1 month postpartum [16], [17]. Thus, the current study was designed to investigate the state of depressive mood and perceived rearing among women using the questionnaire longitudinally from pregnancy through the postpartum. This approach allowed for evaluation of the effects of perceived rearing during pregnancy on onset of postpartum depression as measured by the EPDS, and whether the perception of rearing changes through onset of depressive mood.

In the manner described above, depressive mood and perceived rearing was investigated prospectively over time to: 1) compare and verify the influence of perceived rearing on the pattern of depressive states during pregnancy and the postpartum, and 2) to examine whether the evaluation of perceived rearing is affected by depressive mood at the time of evaluation.

## Materials and Methods

### Ethics Statement

The study was explained to the participants both verbally and in writing, and written consent was obtained from all participants. Study protocol was approved by the Ethics Committees of Nagoya University Graduate School of Medicine and the institutes and hospitals involved, and the study itself was conducted in conformity with the established ethical standards of all institutions.

### Participants

Female participants were recruited from prenatal classes for pregnant women (starting before the 25th week of pregnancy) at two obstetric hospitals located in central Nagoya, Japan (with a population of approximately 2 million) between August 2004 and October 2010. The participants were randomly sampled at these institutions. The follow-up period was 6 months after delivery [16]. Mothers with current or past histories of mental problems were excluded from the study, as well as mothers with children born before the 36th week of gestation.

A total of 643 Japanese women (20 years and older) agreed to participate in the study. Of this total, 467 (72.6%) completed the EPDS at all time points (T1: early pregnancy before the 25th week of pregnancy; late pregnancy: around week 36 of pregnancy; and T2∶1 month after delivery), and 558 (86.8%) completed the PBI without omission at T1. This amounted to 448 women (69.7%) completing the EPDS at all time points and the PBI without omission at T1 (mean age 31.8±4.2, range 22–44 years of age).

### Measures

#### The Edinburgh Postnatal Depression Scale (EPDS)

The EPDS is a self-report questionnaire consisting of ten items to evaluate depressive states after delivery [15], employing a four-point assay (0–3 points) yielding a total score ranging from a minimum of 0 to a maximum of 30. The scale focuses on the cognitive and affective aspects of depression, excluding the physical symptoms such as loss of appetite. The reliability and validity of the scale in a Japanese general population sample has been reported at 75% and 93% (cut-off ≥9 points), respectively [18]. It’s efficacy as a screening scale for depression during pregnancy has also been demonstrated [19]. Thus, a score of 9 points on the EPDS was selected as the cut-off point for screening the depression groups in this study. This screening was repeated three times: in early pregnancy before the 25th week: T1), late pregnancy (around week 36), and at 1 month after delivery (T2).

#### The Parental Bonding Instrument (PBI)

The PBI was used to evaluate perceived rearing [20]. The PBI is a self-report questionnaire evaluating perception of how one was raised by recalling the parents’ child-rearing attitudes before one reached 16 years of age. The scale consists of 25 items each for the father figure, and the mother figure. The child-rearing attitudes are evaluated on a four-point scale (0–3 points), regarding 12 care category items and 13 overprotection category items. Possible scores for the care category ranges from 0 to 36 points– higher scores indicating more acceptive and more affectionate rearing attitudes on the part of the parents; a lower score indicating apathy and rejection. The overprotection category ranges from 0 to 39 points–higher scores indicating the parents’ rearing attitudes as being overprotective and excessively interfering; a lower score indicating respect for self-subsistence. Overall, the evaluation consists of four categories: paternal care, paternal overprotection, maternal care, and maternal overprotection, and scores were obtained for each category. Kitamura and Suzuki translated the PBI into Japanese incorporating back-translation in 1993 to confirm the validity of this Japanese edition [21].

As part of this study, the Japanese version of the PBI was administered in early pregnancy (T1) and again at 1 month postpartum (T2) in some participants to examine whether the PBI scores were subject to change in accordance with transition in EPDS scores. A point of note is the discrepancy in number of subjects regarding PBI scores at T1 and T2, due to the fact that the PBI was not initially assessed at T2 in launching the cohort study.

**Table 1:** Participant profiles in the four initial subject categories.

	period							
	pregnancy		postpartum					
groups	T1	late	T2	n	%	age		p-value^a^
						mean	SD	
non depressive (ND) group	–	–	–	321	71.7	31.9	4.0	0.97
postpartum depressive (PD) group	–	–	+	49	10.9	32	3.9	
temporary gestational depressive (TG) group	+	+	–	50	11.2	31.7	5.2	
	+	–	–					
	–	+	–					
continuous depressive (CD) group	+	+	+	28	6.3	31.5	4.3	
	+	–	+					
	–	+	+					
All				448	100	31.8	4.2	

T1: early pregnancy (before 25 weeks).

late: late pregnancy (around 36 weeks).

T2: postpartum (1 month).

+: EPDS >8, − : EPDS <9.

a: ANOVA was used to test the mean differences of age within the four groups.

### Study Design

Pregnant women participating in prenatal classes were provided detailed verbal and written explanations about the nature and scope of this study. Subjects consenting to partake in the study were given a demographic questionnaire, the EPDS, and the PBI, with instructions to complete the forms at home, and returned by mail.

In line with a preceding analysis of the EPDS results by Ishikawa *et al.* [16], the 448 subjects of this study were initially divided according to the timing of exhibiting depression into 4 groups as follows: 1) a non depressive (ND) group, with EPDS scores below the cut-off point at all time points; 2) a postpartum depressive (PD) group, with EPDS scores exceeding the cut-off point only at 1 month postpartum; 3) a temporary gestational depressive (TG) group, with EPDS scores exceeding the cut-off point only during pregnancy; and 4) a continuous depressive (CD) group, exhibiting EPDS scores above the cut-off point during both pregnancy and the postpartum (Table 1). The merit of classifying the participants into these four groups was that it enabled distinction between those who did not present with symptoms of depression during pregnancy (ND and PD groups) from those who did (TG and CD groups). The TG and CD groups were excluded from subsequent analyses to preclude subjects with possibly unidentified mood disorders, including MDD or bipolar disorder, as the present study did not employ structured interviews (such as the Structured Clinical Interview for DSM Disorders) to rule out such mood disorders.

As the first step in this study, difference in PBI scores at T1 were compared between the ND and PD groups to examine whether levels of PBI in pregnant women was an indicator for risk of postpartum depression. Thus, the remaining 370 participants (ND, n = 321; PD, n = 49) were selected as the subjects for the current analysis.

Next, association in the change between EPDS and PBI scores was examined in the 80 subjects submitting PBI scores at T2 in addition to T1 (ND, n = 63; PD, n = 17) to evaluate whether PBI scores increase with increase in EPDS scores from T1 to T2–i.e., measurement of the state dependency of PBI scores. As noted previously, there is a discrepancy in the number of subjects with PBI scores at T1 (n = 448) and T2 (n = 80), due to the fact that the PBI was not administered at T2 at the start of this cohort study.

### Statistical Analysis

Analysis of variance (ANOVA) was used to test the mean difference of age within the four initial EPDS groups (Table 1). The Student’s t-test was used to compare PBI scores between the ND and PD groups at T1 (Table 2). The paired t-test was used to compare change in EPDS score and PBI score from T1 to T2 in the ND and PD groups (Tables 3 and 4). Correlation between changes in EPDS and PBI scores from T1 to T2 was examined based on Pearson’s coefficients (r) within the ND and PD groups (Table 5). Significance levels were set at p<0.05. All p-values were two-tailed. IBM SPSS Statistics Version 19 (IBM Japan, Tokyo) was used in all analyses.

**Table 2:** PBI scores in the ND and PD groups at T1.

	ND group		PD group		^a^P-value
	n = 321		n = 49		
Category of PBI	mean	SD	mean	SD	
Paternal care	24.6	8	22.3	9.5	0.07
Paternal overprotection	11.1	6.9	12.2	9.6	0.33
Maternal care	29.6	7.2	28.5	8.1	0.31
Maternal overprotection	10.9	7.5	10.2	7.5	0.71

PBI: Parental Bonding Instrument.

T1: early pregnancy (before 25 weeks).

ND group: non depressive group.

PD group: postpartum depressive group.

SD: standard deviation.

a: Student’s t-test between the ND and PD group.

## Results

Participant profiles are shown in Table 1. No significant differences in age were found across the initial four subject groups (p = 0.97, ANOVA) (Table 1). There were no significant differences in any PBI category (paternal care, paternal overprotection, maternal care, and maternal overprotection) between the non depressive (ND) group and postpartum depressive (PD) group at T1 (Table 2). Changes in EPDS scores in the ND and PD groups from T1 to T2 are shown in Table 3. The EPDS scores did not change significantly from T1 to T2 in the ND group (p = 0.45), but significantly increased in the PD group (p<0.0001). Changes in PBI scores in the ND and PD groups are shown in Table 4. In both ND and PD groups, there were no significant differences in scores for paternal care, paternal overprotection, and maternal overprotection between T1 and T2 (all p>0.05). However, the scores for maternal care significantly increased in the ND group (p = 0.008) while significantly decreasing in the PD group (p = 0.044). A weak but significant correlation was confirmed between the change in maternal care score and change in EPDS score in the ND and PD groups (r = −0.28, p = 0.013) (Table 5). No correlation was noted between the EPDS and any of the other PBI scores (all p>0.05) (Table 5).

**Table 3:** EPDS scores in the ND and PD groups.

		T1		T2		^a^P-value
	n	mean	SD	mean	SD	
ND group	321	2.8	2.4	2.6	2.3	0.45
PD group	49	4.0	2.2	12.2	3.4	<0.0001
All	370	2.9	2.4	3.9	4.1	

EPDS: Edinburgh Postnatal Depression Scale.

ND group: non depressive group.

PD group: postpartum depressive group.

T1: early pregnancy (before 25 weeks).

T2: postpartum (1 month).

SD: standard deviation.

a: Paired t-test.

**Table 4:** PBI scores in the ND and PD groups.

			T1		T2		^a^P-value
	group	n	mean	SD	mean	SD	
Paternal care	ND group	63	25.3	7.6	26.1	7.4	0.13
	PD group	17	21.2	10.6	20.3	10.8	0.15
Paternal overprotection	ND group	63	12.1	6	11.4	6.3	0.17
	PD group	17	10.5	7.8	10.7	6.5	0.85
Maternal care	ND group	63	29.7	6.8	30.7	6.3	**0.008**
	PD group	17	27.8	10.6	26.5	10.8	**0.044**
Maternal overprotection	ND group	62	11.6	7.1	11.3	7.6	0.54
	PD group	17	10.7	7.6	11.9	8.7	0.37

PBI: Parental Bonding Instrument.

ND group: non depressive group.

PD group: postpartum depressive group.

T1: early pregnancy (before 25 weeks).

T2: postpartum (1 month).

SD: standard deviation.

a: Paired t-test between T1 and T2.

**Table 5:** Correlations between PBI and EPDS score changes from T1 to T2 in the ND and PD groups.

	n	r	P-value
Parental care	80	−0.21	0.06
Parental overprotection	80	−0.02	0.85
Maternal care	80	−0.28	**0.013**
Maternal overprotection	79	0.04	0.69

PBI: Parental Bonding Instrument.

EPDS: Edinburgh Postnatal Depression Scale.

T1: early pregnancy (before 25 weeks).

T2: postpartum (1 month).

ND group: non depressive group.

PD group: postpartum depressive group.

r: Pearson’s r.

## Discussion

### Principal Findings

The present study is the first prospective investigation on the relationship between depressive mood and perceived rearing through pregnancy and the postpartum in a cohort of pregnant Japanese women. The findings revealed absence of any significant difference in perceived rearing between the ND and PD groups during pregnancy. This appears to indicate that perceived rearing is not a strong risk factor for postpartum depression as measured by the EPDS. On the other hand, significant correlation was found between changes in maternal care score and EPDS score from T1 to T2 in the ND and PD groups, suggesting state dependency of the PBI maternal care score.

Many studies have examined the relationship between perceived rearing and postpartum depression. The results have been inconsistent, although this might be due at least in part to the inclusion of subjects with persistent depression in the ‘postpartum depression’ group in those previous studies. We observed different sequences over time regarding the depressive state possibly indicating a different etiology for depression during pregnancy and the postpartum. The possibility of such differences were not addressed in the previous studies conducted on postpartum depression groups including women who would have belonged to a CD (continuous depression) group had that distinction been made, not to mention women falling into a TG (temporary gestational depressive) group who may have suffered from mood disorders including major depressive disorder or bipolar disorder. In the present study, analysis of the EPDS results were conducted excluding such subjects (groups TG and CD) from the ND and PD groups. This exclusion is one of the strengths of our study. In addition, the sample size used in the current study was large and bias effects were relatively small given the prospective design. Moreover, all subjects were Japanese, making the genetic and cultural confounders negligible.

Depressive symptoms were observed in 28.3% of the initial sample of 448 subjects at least one of the three time points between early pregnancy and 1 month postpartum. Prevalence during pregnancy (17.5%) and the postpartum (17.2%) was similar to that reported in previous studies [22], [23], [24], [25]. Regarding the course of depressive state, only 10.9% of the subjects were examined in this analysis as constituting the PD group, i.e., subjects exhibiting depressive states measured by the EPDS only after delivery.

As a result of this maneuver, no significant differences were found in mean scores for each PBI category between the ND and PD groups at T1. This result is inconsistent with previous studies suggesting a relationship between perceived rearing and postpartum depression. A probable cause for this discrepancy is the inclusion of subject groups with different psychopathologies in the previous studies. The previous studies using the PBI have revealed relationships between onset of eating disorders [26]/obsessive-compulsive disorder [27]/borderline personality disorder [26]/depression [28], [29], [30], [31], and parents’ inappropriate rearing styles characterized by low care and/or high overprotection. It is well known that patients with these psychiatric disorders frequently exhibit depression during the course of illness. Thus, datasets including such subjects may have affected the results of such previous investigations.

Our findings also showed significant increase in maternal care scores in the ND group, in contrast to its significant decrease in the PD group. In addition, significant correlation was found between maternal care and EPDS score changes from T1 to T2 in both ND and PD groups. This may be due to the experience of childbirth and depressive state possibly distorting the perception of rearing. As pointed out in previous studies, depressed patients exhibit a tendency for negative recollections [32], [33]. A previous study has reported correlation of the PBI score with the Beck Depression Inventory [34], although most longitudinal studies have indicated consistency of the PBI regardless of fluctuations in mood [9], [10], [11], [12], [13], [35], [36]. Our results were in line with such studies demonstrating the state dependency of perceived rearing [31], [37], [38].

### Limitations

Lastly, this study has its limitations. The mental states of the subjects were evaluated only with self-administered questionnaires (EPDS and PBI). The EPDS is usually used as a screening scale for depression during the perinatal period and there is no consensus of its validity as an index for indicating the degree of depression. As we adopted 9 as the EPDS cutoff (sensitivity: around 77%, specificity: 69%, area under the ROC curve: 75%), 25% of the subjects may not have been correctly classified [18], [39]. Thus, the initial classification of subjects into four groups (ND, PD, TG and CD) by the EPDS might be different from the classification by structured clinical interviews such as the SCID. Additionally, histories regarding mood disorders before pregnancy were not assessed, and even after excluding the TG and CD groups, it is possible the ND and PD groups might still include subjects with, for example, bipolar disorder [40]. In future, it may be helpful to assess histories of mood disorders using diagnostic tools such as the SCID. It should also be useful to take into account the effects of repetitive measurement using the same questionnaire during a short period of time, as well as the effects of various psychosocial factors, including the relationship with partners, socioeconomic factors, relevant demographical data (number of family members, age and gender of children) because these factors are likely to influence the results. Additionally, previous studies have shown significant changes in physiological and psychological functions during the different trimesters of pregnancy [41], [42]. Such changes could predispose women to develop depression to a certain degree, suggesting a time-specific threshold might be appropriate [43]. However, as these thresholds have not been validated in the Japanese population, a single cutoff score was adopted for this study. And with regards to the multiple comparison problem, we did not adjust the threshold of significance in the current study. After the Bonferonni correction, several p-values were not under the threshold of significance. Thus, our results need to be replicated in independent studies.

### Conclusions

We examined how the experience of being raised as evaluated by the PBI influences the onset of postpartum depressive mood, and how the onset of depressive state influences the recall of perceived rearing. Several findings were obtained through this study: 1) no significant differences were noted in perceived rearing between ND and PD groups; 2) the PBI score for maternal care increased significantly in the ND group, while significantly decreasing in the PD group; 3) significant correlation was noted between changes in the EPDS and the PBI maternal care score from T1 to T2. This study suggests that perceived rearing during pregnancy is apparently not a strong risk factor for postpartum depression as measured by the EPDS, while demonstrating the state dependency of perceived maternal care assessed by the PBI.
